# Supplementary material for: A Likelihood Approach for Real-Time Calibration of Stochastic Compartmental Epidemic Models
Source: PLoS Comput Biol. 2017 Jan 17;13(1):e1005257. doi: 10.1371/journal.pcbi.1005257 (PMC5240920; doi:10.1371/journal.pcbi.1005257)
Supplement: S1 File — (TAR.GZ) [file pcbi.1005257.s014.tar.gz › HSPH_Online-SI-Revision/EnKF07/n10-extreme/n10-extreme_EnKF_table-8.pdf]

1	R0	{2.68692, 12.5884}	{2.20136, 10.1939}	{{0.797087, 2.02125}, {3., 16.9593}}	{27.8712, 39.4503}	inf	21.	2354.33	{555.022, 4017.14}	11116.5	1.91136	newly	13.	2404.78	{435., 6255.}	18404.	2.04821	Reff	2.6759	1.36382	{0.388247, 2.21906}	50.2317	ar	9089.	12861.3	{7615., 17316.}	47.1263
2	R0	{1.79802, 12.5412}	{2.11379, 4.49129}	{{1.19028, 2.67875}, {3., 5.15154}}	{45.3564, 64.5782}	inf	125.	1699.15	{566.286, 5425.44}	1261.63	0.965291	newly	89.	1840.63	{656., 5505.}	1970.53	1.11042	Reff	1.74857	0.374053	{0.0368156, 0.793586}	80.4099	ar	7412.	16610.2	{8498., 26269.}	126.314
3	R0	{2.6174, 10.1105}	{1.73198, 7.60804}	{{0.381408, 2.00079}, {3., 16.1415}}	{41.878, 51.46}	inf	4.	700.123	{119.774, 1362.34}	17403.1	2.0558	newly	3.	2281.5	{186., 7107.}	75950.	2.46763	Reff	2.61609	1.55511	{0.312447, 2.8823}	43.9446	ar	9109.	6656.95	{1501., 10525.}	36.7965
4	R0	{2.27532, 14.3149}	{2.20763, 4.24687}	{{0.998504, 2.67856}, {3., 5.12334}}	{36.8108, 70.3325}	inf	174.	1601.39	{910.469, 5224.31}	822.989	0.839165	newly	107.	1653.28	{686., 5368.}	1447.7	1.03763	Reff	2.20251	0.322199	{0.0651613, 0.485787}	85.3713	ar	8478.	15358.8	{6321., 26118.}	91.7414
5	R0	{2.96649, 18.0674}	{2.13809, 6.04014}	{{0.857933, 2.15979}, {3., 10.0675}}	{28.9644, 66.5688}	inf	107.	2904.27	{769.557, 5093.64}	2619.55	1.34825	newly	64.	3622.68	{1157., 6531.}	5564.91	1.61325	Reff	2.92051	1.11313	{0.366529, 2.16716}	61.8857	ar	9476.	13152.7	{8255., 19697.}	47.526
6	R0	{2.94778, 16.0002}	{2.2648, 10.0841}	{{0.886946, 2.02166}, {3., 16.9487}}	{24.8347, 40.3303}	inf	56.	2454.18	{361.213, 4406.57}	4290.84	1.49642	newly	41.	2632.6	{345., 6127.}	6330.83	1.58365	Reff	2.92125	1.35428	{0.276714, 2.21918}	53.8808	ar	9321.	12828.8	{6643., 17287.}	46.3701
7	R0	{2.17562, 11.7184}	{2.22788, 9.24794}	{{0.59125, 2.10791}, {3., 14.905}}	{35.8544, 37.0619}	inf	23.	1930.26	{684.464, 4119.99}	8292.43	1.81283	newly	18.	2474.05	{456., 7173.}	13644.7	1.92523	Reff	2.16844	1.55031	{0.256186, 2.67735}	36.5875	ar	8345.	11948.9	{5435., 18435.}	54.6509
8	R0	{2.7904, 17.8966}	{2.54416, 7.03389}	{{2.12865, 4.05921}, {3., 10.8768}}	{16.0357, 60.7589}	inf	98.	2029.12	{640.378, 3464.41}	1971.36	1.17785	newly	46.	2216.2	{588., 5853.}	4717.83	1.50067	Reff	2.74575	0.948472	{0.344255, 2.15334}	65.4568	ar	9285.	15162.	{8888., 22280.}	67.2886
9	R0	{2.51574, 16.376}	{2.54426, 7.03708}	{{2.12992, 4.02053}, {3., 10.8202}}	{17.3454, 57.2713}	inf	112.	2028.04	{643.172, 3488.41}	1712.12	1.12252	newly	55.	2216.45	{583., 5851.}	3929.91	1.42345	Reff	2.47171	0.948993	{0.342606, 2.14746}	61.6059	ar	8937.	15200.3	{8853., 22373.}	73.4092
10	R0	{2.56405, 18.1787}	{2.57859, 6.9921}	{{2.13218, 4.02036}, {3., 10.8673}}	{16.7755, 61.5587}	inf	124.	2087.17	{645.227, 4267.34}	1584.79	1.09546	newly	69.	2250.94	{585., 5852.}	3162.23	1.33262	Reff	2.51226	0.919108	{0.390726, 2.14106}	63.4151	ar	9005.	15055.5	{8395., 22271.}	70.459
11	R0	{2.475, 17.1709}	{2.35182, 5.97475}	{{1.49038, 2.4551}, {3., 10.0794}}	{25.4907, 65.2042}	inf	144.	305.942	{174.366, 461.316}	118.148	0.215977	newly	77.	606.57	{89., 2377.}	691.364	0.505832	Reff	2.41461	0.614486	{0.239289, 1.47621}	74.6231	ar	8870.	18051.9	{16754., 21541.}	103.516
12	R0	{2.14974, 11.8619}	{2.62868, 7.47077}	{{2.13641, 4.19435}, {3., 14.6866}}	{24.611, 44.3465}	inf	45.	2076.91	{636.929, 3492.65}	4517.63	1.51863	newly	37.	2020.67	{575., 4884.}	5362.51	1.55838	Reff	2.13147	0.895858	{0.344701, 2.04116}	58.5626	ar	8414.	14890.4	{9536., 22073.}	80.449
13	R0	{2.20385, 14.4767}	{2.50215, 5.56512}	{{2.02733, 4.20559}, {3., 7.79973}}	{25.7202, 61.5581}	inf	171.	239.258	{34.5425, 256.987}	94.4154	0.327359	newly	105.	268.86	{23., 517.}	176.476	0.370316	Reff	2.13685	0.636697	{0.181549, 1.80741}	70.204	ar	8347.	18478.2	{16558., 24414.}	121.376
14	R0	{2.35671, 13.8357}	{2.18056, 6.26883}	{{0.858292, 2.15966}, {3., 10.0688}}	{32.9101, 55.9855}	inf	81.	2765.6	{771.117, 5165.07}	3319.83	1.41949	newly	53.	3368.22	{1142., 6999.}	6258.08	1.62987	Reff	2.32631	1.108	{0.35118, 2.16839}	52.4329	ar	8719.	13433.	{8276., 19753.}	60.3266
15	R0	{1.91368, 10.1082}	{2.0907, 4.553}	{{1.19014, 2.6783}, {3., 5.14741}}	{41.6928, 56.0541}	inf	100.	1574.21	{586.183, 5204.21}	1476.14	1.03922	newly	95.	1704.42	{672., 5163.}	1696.63	1.06475	Reff	1.87579	0.387626	{0.0388538, 0.792284}	80.7433	ar	7486.	16090.4	{8319., 23286.}	118.441
16	R0	{2.48727, 14.882}	{2.27048, 6.10045}	{{0.862512, 2.15987}, {3., 10.0731}}	{30.9793, 59.5947}	inf	30.	2786.73	{776.77, 5088.03}	9190.57	1.8242	newly	19.	3405.85	{1025., 5991.}	17826.3	2.07679	Reff	2.47508	1.091	{0.35259, 2.15046}	55.9206	ar	8894.	13374.3	{8282., 19762.}	58.0911
17	R0	{2.46795, 13.1915}	{2.21187, 9.09735}	{{0.591296, 2.10799}, {3., 15.2197}}	{30.983, 38.3946}	inf	43.	1876.02	{575.113, 3721.95}	4262.83	1.51225	newly	22.	2557.52	{409., 7990.}	11525.1	1.82556	Reff	2.45487	1.5087	{0.250397, 2.11577}	42.2562	ar	8796.	11706.3	{5366., 18465.}	47.7133
18	R0	{2.96603, 16.7543}	{2.17368, 6.19179}	{{0.858668, 2.15975}, {3., 10.0693}}	{27.7679, 63.2685}	inf	59.	2814.44	{771.285, 5374.89}	4674.82	1.5511	newly	37.	3395.01	{1154., 6934.}	9077.7	1.7835	Reff	2.93429	1.09765	{0.350373, 2.16749}	62.5925	ar	9394.	13370.3	{8104., 19731.}	50.8846
19	R0	{2.25216, 9.90465}	{2.22774, 9.25106}	{{0.590515, 2.1081}, {3., 14.9237}}	{34.4373, 40.6083}	inf	7.	1930.23	{683.796, 4121.58}	27474.8	2.32979	newly	5.	2472.61	{457., 7151.}	49352.2	2.48259	Reff	2.24946	1.55092	{0.256037, 2.67897}	37.9802	ar	8631.	11967.4	{5360., 18426.}	51.1942
20	R0	{2.70837, 18.1923}	{2.62866, 7.47255}	{{2.13641, 4.20325}, {3., 14.6847}}	{15.0029, 58.9438}	inf	85.	2078.06	{635.119, 3492.99}	2349.27	1.26114	newly	45.	2021.21	{571., 4840.}	4393.31	1.47823	Reff	2.67506	0.896032	{0.34466, 2.04526}	66.5042	ar	9183.	14904.3	{9607., 22250.}	66.7493
21	R0	{2.30288, 15.5877}	{2.03097, 4.87941}	{{0.162159, 0.880705}, {3., 5.9719}}	{40.8684, 68.6971}	inf	145.	380.293	{99.1996, 603.927}	173.419	0.37625	newly	76.	396.05	{83., 808.}	426.592	0.590166	Reff	2.2437	0.272208	{0.0237062, 0.459475}	87.8679	ar	8674.	16039.6	{15311., 17014.}	86.2344
22	R0	{2.82208, 10.7544}	{1.71244, 7.62459}	{{0.331799, 2.00079}, {3., 16.1394}}	{42.739, 49.7194}	inf	4.	706.841	{119.985, 1365.47}	17571.	2.06161	newly	9.	2261.04	{165., 7191.}	25023.1	1.97984	Reff	2.82039	1.5348	{0.296968, 2.88194}	46.7259	ar	9333.	6574.86	{1550., 10547.}	37.3833
23	R0	{1.69923, 10.1366}	{2.36911, 5.17724}	{{1.78353, 1.07899}, {3., 13.1516}}	{42.7148, 59.9377}	inf	114.	2311.89	{505.022, 5474.86}	1929.68	1.15148	newly	88.	2749.65	{475., 7416.}	3025.99	1.28793	Reff	1.65454	0.640581	{0.173394, 1.34449}	61.7013	ar	7055.	15105.1	{7152., 24698.}	117.795
24	R0	{2.64944, 19.054}	{2.09982, 4.05224}	{{0.150492, 0.875647}, {3., 5.95727}}	{34.7945, 78.7329}	inf	333.	474.161	{89.8285, 887.446}	85.1826	0.288153	newly	143.	541.93	{73., 1080.}	299.224	0.4889	Reff	2.50266	0.32687	{0.020891, 0.459389}	87.082	ar	9147.	16517.6	{15368., 18387.}	83.2944
25	R0	{2.53191, 10.9214}	{2.63677, 7.82301}	{{2.13445, 4.07922}, {3., 14.6954}}	{16.5647, 39.8251}	inf	6.	2150.69	{650.33, 3499.33}	35744.8	2.43386	newly	9.	2131.87	{609., 5166.}	23587.4	2.21023	Reff	2.52811	0.917318	{0.34506, 2.09697}	63.7153	ar	9009.	14870.5	{10353., 21302.}	68.4028
26	R0	{1.89155, 6.76944}	{2.20704, 9.09916}	{{0.591885, 2.10814}, {3., 13.2208}}	{46.5652, 65.974}	inf	21.	1900.07	{683.662, 4358.15}	8947.96	1.81815	newly	28.	2408.24	{454., 7727.}	8501.57	1.69222	Reff	1.88512	1.4926	{0.248687, 2.11725}	32.6023	ar	7348.	12020.6	{5195., 19650.}	74.295
27	R0	{2.63149, 15.6979}	{2.27687, 5.99}	{{0.863064, 2.15981}, {3., 10.072}}	{28.6001, 62.5386}	inf	43.	2834.53	{814.262, 5140.9}	6494.42	1.69847	newly	23.	3564.42	{1161., 6073.}	15397.5	2.03037	Reff	2.61465	1.14646	{0.352265, 2.17589}	56.1525	ar	9104.	13456.6	{8287., 19685.}	56.0555
28	R0	{2.85141, 12.6992}	{1.70918, 8.03122}	{{0.332067, 2.00079}, {3., 16.2715}}	{42.6384, 50.8025}	inf	11.	686.602	{120.616, 1366.1}	6141.84	1.61693	newly	9.	2037.19	{157., 5405.}	22535.9	1.94433	Reff	2.84685	1.53035	{0.273449, 2.90406}	47.2395	ar	9317.	6598.65	{1555., 10593.}	37.6835
29	R0	{2.54244, 12.9085}	{2.41257, 5.36859}	{{1.67657, 1.06457}, {3., 13.1907}}	{23.1048, 59.5308}	inf	35.	2367.12	{494.268, 5467.9}	6663.2	1.62723	newly	22.	2553.52	{479., 6314.}	11506.9	1.8424	Reff	2.52846	0.587097	{0.246063, 1.04997}	76.7804	ar	9034.	15604.7	{10610., 25572.}	76.8032
30	R0	{2.10982, 11.7636}	{2.38746, 5.50632}	{{1.67188, 1.25427}, {3., 13.1752}}	{30.1001, 58.4966}	inf	84.	2423.19	{498.039, 5478.21}	2785.85	1.28663	newly	65.	2786.09	{495., 7980.}	4186.91	1.42659	Reff	2.07501	0.681235	{0.218238, 1.3455}	67.253	ar	8224.	15542.5	{7570., 25637.}	94.9137
31	R0	{2.76312, 15.5396}	{2.29006, 10.8931}	{{1.37335, 6.37095}, {3., 16.9442}}	{25.522, 36.3462}	inf	70.	2551.82	{361.317, 5322.16}	3553.11	1.41017	newly	46.	2446.51	{156., 4962.}	5227.41	1.48599	Reff	2.73438	1.39048	{0.301275, 2.21903}	50.2074	ar	9220.	12873.9	{6537., 17617.}	48.5227
32	R0	{2.21727, 15.5439}	{2.142, 9.15543}	{{0.492976, 5.93767}, {3., 14.543}}	{43.9041, 41.0995}	inf	175.	458.091	{312.632, 690.503}	165.249	0.341641	newly	111.	346.85	{117., 406.}	216.315	0.359464	Reff	2.14809	0.397047	{0.0520207, 0.669356}	81.5163	ar	8359.	17931.4	{16911., 19526.}	114.516
33	R0	{2.72912, 10.8159}	{2.27025, 9.74202}	{{1.36968, 2.02103}, {3., 16.9411}}	{25.6464, 39.6494}	inf	10.	2401.77	{493.149, 4203.14}	23921.1	2.22245	newly	16.	2579.99	{408., 6383.}	16028.9	1.99559	Reff	2.72393	1.37953	{0.399909, 2.219}	50.2708	ar	9196.	12812.8	{7740., 17235.}	46.2391
34	R0	{2.20928, 10.1308}	{1.7104, 8.12613}	{{0.262328, 2.00079}, {3., 17.4837}}	{45.4941, 52.5823}	inf	22.	722.225	{132.58, 1455.49}	3185.88	1.32794	newly	14.	2053.96	{93., 5715.}	14572.3	1.73848	Reff	2.20155	1.50351	{0.262302, 2.71698}	46.0653	ar	8388.	6589.5	{1457., 10624.}	40.9275
35	R0	{2.10103, 15.2673}	{2.40659, 5.15627}	{{1.66954, 1.13906}, {3., 13.1683}}	{30.6592, 66.3283}	inf	193.	2410.95	{503.62, 5480.86}	1151.68	0.941811	newly	113.	2830.38	{485., 7938.}	2406.09	1.20476	Reff	2.038	0.666368	{0.19877, 1.3447}	67.3028	ar	8264.	15577.3	{7589., 25618.}	94.4596
36	R0	{2.74914, 19.2775}	{1.99523, 4.43508}	{{0.171412, 0.882812}, {3., 5.96498}}	{35.271, 76.9935}	inf	143.	414.825	{100.709, 584.708}	201.603	0.387886	newly	67.	471.59	{93., 901.}	609.866	0.679646	Reff	2.68618	0.285009	{0.0238525, 0.458945}	89.3898	ar	9301.	16360.9	{15329., 17429.}	76.8841
37	R0	{2.78161, 16.0136}	{2.58819, 7.57857}	{{2.13328, 4.17767}, {3., 14.6931}}	{14.9616, 52.9738}	inf	38.	2097.42	{642.438, 3499.1}	5420.98	1.59836	newly	25.	2040.4	{599., 4973.}	8061.6	1.72976	Reff	2.76659	0.891794	{0.31905, 2.09622}	67.7656	ar	9240.	14917.	{10355., 22072.}	65.6714
38	R0	{2.4454, 8.33501}	{2.21351, 9.13353}	{{0.590346, 2.1081}, {3., 14.9247}}	{31.4923, 46.9064}	inf	3.	1980.42	{683.796, 4356.6}	65913.9	2.70815	newly	9.	2592.39	{456., 7979.}	28704.3	2.23828	Reff	2.44369	1.51623	{0.255953, 2.67926}	42.5486	ar	8853.	11761.	{5354., 18437.}	48.4439
39	R0	{2.9154, 12.3861}	{2.21348, 9.13407}	{{0.590194, 2.10814}, {3., 14.9285}}	{26.7478, 35.9672}	inf	14.	1980.41	{683.662, 4356.58}	14045.8	2.03921	newly	12.	2587.01	{443., 7945.}	21458.4	2.11114	Reff	2.91044	1.51634	{0.255919, 2.6796}	48.3906	ar	9371.	11758.8	{5474., 18408.}	43.7215
40	R0	{2.40923, 15.1963}	{2.47976, 6.93237}	{{2.12818, 3.964}, {3., 10.0526}}	{15.8693, 54.7928}	inf	149.	1931.08	{636.476, 3257.62}	1199.86	0.99445	newly	86.	2182.83	{595., 5200.}	2440.01	1.2285	Reff	2.34731	0.999007	{0.380936, 2.19826}	57.4581	ar	8735.	14841.1	{8471., 21208.}	73.1755
41	R0	{2.63137, 15.238}	{2.45004, 4.64787}	{{1.69503, 0.940075}, {3., 13.1423}}	{21.7407, 69.577}	inf	88.	2313.61	{496.946, 5472.33}	2530.67	1.2488	newly	67.	2779.03	{482., 7904.}	4047.81	1.40775	Reff	2.59269	0.636881	{0.20165, 1.34509}	75.4355	ar	9150.	15282.6	{7554., 25569.}	74.4864
42	R0	{2.48769, 19.4593}	{2.19572, 9.33527}	{{0.489736, 5.98367}, {3., 14.5215}}	{35.9562, 52.0267}	inf	353.	464.31	{313.273, 313.273}	65.3983	0.254371	newly	145.	350.02	{111., 319.}	163.724	0.399788	Reff	2.34714	0.374226	{0.0500584, 0.667367}	84.0561	ar	8932.	17812.5	{16865., 18590.}	99.4234
43	R0	{2.61199, 10.214}	{1.73198, 7.60804}	{{0.381408, 2.00079}, {3., 16.1415}}	{41.8777, 51.0804}	inf	1.	700.123	{119.774, 1362.34}	69912.3	2.65786	newly	2.	2281.5	{186., 7107.}	113975.	2.64372	Reff	2.61147	1.55511	{0.312447, 2.8823}	43.9161	ar	9061.	6656.95	{1501., 10525.}	36.896
44	R0	{2.50994, 18.3382}	{2.26971, 5.30192}	{{1.48728, 2.47134}, {3., 10.0821}}	{25.972, 71.0881}	inf	194.	447.136	{168.732, 997.405}	154.454	0.257883	newly	107.	880.77	{90., 3934.}	733.729	0.557104	Reff	2.42761	0.713368	{0.242542, 1.50121}	70.7343	ar	8827.	18842.6	{16822., 24802.}	113.465
45	R0	{2.44283, 16.8338}	{2.36835, 5.20026}	{{1.78078, 1.11863}, {3., 13.1813}}	{23.0622, 69.1082}	inf	169.	2410.15	{511.797, 5479.32}	1328.83	1.00287	newly	105.	2804.07	{496., 7401.}	2571.76	1.22536	Reff	2.37394	0.642042	{0.17196, 1.34362}	72.9546	ar	8878.	15250.2	{7126., 25561.}	79.1631
46	R0	{2.51815, 18.1462}	{2.02198, 4.23784}	{{0.16229, 0.886019}, {3., 5.96538}}	{38.0734, 76.6461}	inf	163.	474.356	{128.653, 752.27}	201.608	0.386413	newly	80.	518.44	{103., 1074.}	552.525	0.641694	Reff	2.44689	0.265504	{0.0249417, 0.45982}	89.1493	ar	8978.	16446.	{15437., 18015.}	85.8428
47	R0	{2.88885, 16.8987}	{2.40792, 5.36751}	{{1.67712, 1.05375}, {3., 13.1974}}	{20.4953, 68.2372}	inf	49.	2340.9	{494.249, 5468.69}	4677.9	1.47698	newly	30.	2539.27	{484., 6266.}	8364.83	1.70211	Reff	2.86458	0.586666	{0.261622, 1.04907}	79.52	ar	9337.	15565.9	{10033., 25641.}	71.0825
48	R0	{2.58709, 13.8786}	{2.20134, 10.1937}	{{0.797509, 2.02167}, {3., 16.9579}}	{28.4849, 39.5461}	inf	46.	2354.46	{554.965, 4017.4}	5024.27	1.59323	newly	26.	2400.62	{427., 6064.}	9139.92	1.77032	Reff	2.57002	1.36386	{0.388185, 2.21906}	48.6746	ar	9146.	12865.6	{7593., 17282.}	46.3527
49	R0	{2.42959, 10.7132}	{2.21351, 9.13353}	{{0.590346, 2.1081}, {3., 14.9247}}	{31.6972, 39.0873}	inf	18.	1980.42	{683.796, 4356.6}	10902.3	1.93	newly	13.	2592.39	{456., 7979.}	19841.5	2.07858	Reff	2.42424	1.51623	{0.255953, 2.67926}	42.2803	ar	8863.	11761.	{5354., 18437.}	48.3509
50	R0	{2.60513, 13.2796}	{2.20742, 5.43757}	{{0.865282, 2.1599}, {3., 10.071}}	{31.0301, 59.3526}	inf	24.	2706.61	{808.722, 5140.61}	11177.9	1.91638	newly	29.	3637.38	{1173., 8434.}	12442.7	1.93231	Reff	2.59445	1.10907	{0.351321, 2.17809}	57.2521	ar	9095.	13156.8	{7550., 20035.}	56.3532
